# (Dys)regulation of the Immune System in Parkinson’s Disease: Methodologies, Techniques, and Key Findings from Human Studies

**DOI:** 10.14336/AD.2024.1163

**Published:** 2025-03-26

**Authors:** Christina M. Lill, Alessia di Flora, Leonardo A. Sechi, Frederico C. Pereira, Nicoleta Moisoi, Neda Nikolovski, Mustafa Aktekin, Yasemin Gursoy-Ozdemir, Laura Deecke, Davide Cossu, Jan Homann, Elena R. Simula, Elif Nedret Keskinoz, Devrim Oz-Arslan, Nuray Bayar Muluk, Milene Gonçalves, Elenamaria Pirovano, Cristoforo Comi, Franca Marino

**Affiliations:** ^1^Institute of Epidemiology and Social Medicine, University of Münster, Münster, Germany;; ^2^Ageing Epidemiology Research Unit, School of Public Health, Imperial College, London, UK; ^3^Center for Research in Medical Pharmacology, University of Insubria, Varese, Italy; ^4^Department of Biomedical Sciences, University of Sassari, Sassari, Italy; ^5^SC Microbiologia e Virologia AOU Sassari, Italy; ^6^Institute of Pharmacology and Experimental Therapeutics & Coimbra Institute for Clinical and Biomedical Research (iCBR), Faculty of Medicine, University of Coimbra, Coimbra, Portugal;; ^7^Center for Innovative Biomedicine and Biotechnology (CIBB), University of Coimbra, Coimbra, Portugal; ^8^Leicester School of Pharmacy, Faculty of Health and Life Sciences, De Montfort University, Leicester, UK; ^9^Department of Immunology, Institute for Biological Research “Siniša Stanković”, National Institute of the Republic of Serbia, University of Belgrade, Belgrade, Serbia;; ^10^Department of Anatomy, School of Medicine, Acıbadem University, Istanbul, Turkey;; ^11^Koç University Research Center for Translational Medicine; Faculty of Medicine, Department of Neurology, Istanbul, Turkey; ^12^Department of Neurology, Juntendo University School of Medicine, Tokyo, Japan; ^13^Department of Biophysics, School of Medicine, Acıbadem University, Istanbul, Turkey;; ^14^Department of Otorhinolaryngology, Faculty of Medicine, Kirikkale University, 71450, Kirikkale, Turkey; ^15^Institute for Interdisciplinary Research, Doctoral Programme in Experimental Biology and Biomedicine (PDBEB), University of Coimbra, Portugal; ^16^Neurology Unit, S. Andrea Hospital, Department of Translational Medicine and Interdisciplinary Research Center of Autoimmune Diseases (IRCAD), University of Piemonte Orientale, Vercelli, Italy.

**Keywords:** Parkinson’s disease;, neurodegeneration, immunology, infection, CD4, CD8

## Abstract

Parkinson’s disease (PD) is the second most common neurodegenerative disorder, characterized by the degeneration of dopaminergic neurons in the midbrain. While PD is typically considered a disorder primarily affecting the central nervous system, there is mounting evidence of cellular dysfunction and PD pathology occurring in the peripheral nervous system, likely preceding central manifestations. In this context, it has become increasingly evident that dysregulation of both the central and the peripheral immune system plays a key role in PD pathogenesis and progression. In this narrative review, we describe and discuss the methodological approaches employed in human studies to investigate immune responses in PD pathogenesis and progression, their main findings and the potential to unveil novel therapeutic avenues. In particular, we present methodologies employed in and insights gained from human genetic studies, techniques utilized to investigate neuroinflammatory processes in post-mortem and living human brains, to investigate the blood-brain barrier, as well as the involvement of peripheral T cells and innate immune cells. Additionally, we elucidate methodologies utilized to explore the roles of mitochondrial dysfunction and infectious diseases in PD. Finally, we address the causes behind conflicting findings in the published literature, which may stem from disparities in sample ascertainment schemes, immunological protocols, and analysis designs. Given these challenges, it becomes imperative to develop methodological guidelines to enhance the validity of immunological studies in PD and facilitate their translation into clinical medicine.

## 1. Introduction

Parkinson’s disease (PD) is second most common neurodegenerative disorder after Alzheimer’s disease (AD) affecting 7-10 million people worldwide [1]. The disease's neuropathological hallmarks include the degeneration of dopaminergic neurons in the substantia nigra pars compacta of the midbrain [2, 3] and the presence of intracellular inclusion bodies known as "Lewy bodies", which contain the α-synuclein protein, in the remaining neurons [4]. The involvement of the midbrain manifests with the classical motor signs and symptoms of PD, including resting tremor, bradykinesia, rigidity, and postural instability [5]. However, Lewy bodies spread throughout the brain as the diseases progresses [6]. Furthermore, beyond dopaminergic neuron loss, the degeneration of other neurotransmitter systems, including serotonin and norepinephrine, significantly contributes to a diverse spectrum of symptoms [7]. For instance, non-motor symptoms such as depression, cognitive impairment and dysautonomia are also common and often proceed the onset of motor symptoms [8].

**Figure 1.**
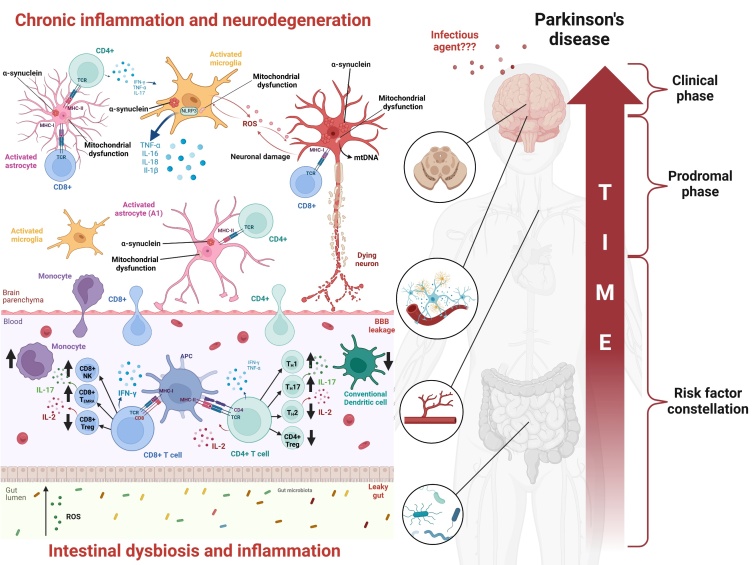
**Immune dysfunction in Parkinson’s disease**. This figure displays the key findings from human studies on the role of immune cells in the pathophysiology of Parkinson’s disease. CD = cluster of differentiation, TCR = T-cell receptor, MHC = major histocompatibility complex, IFN = interferon, TNF = tumor necrosis factor, IL = interleukin, ROS = reactive oxygen species, mtDNA = mitochondrial desoxyribonucleic acid, BBB = blood-brain barrier, NK = natural killer (T cell), TEMRA = terminally differentiated effector memory T cell, Treg = regulatory T cell, T_H_ = T helper, APC = antigen-presenting cell.

There seems to be a close relationship between mitochondrial dysfunction, α-synuclein aggregation, and chronic inflammation in PD. Specifically, dysfunction in mitophagy, a key process for clearing damaged mitochondria, leads to oxidative stress, which promotes α-synuclein accumulation and activates immune signaling pathways (e.g. ref. [9, 10]). Alpha-synuclein itself also localizes to mitochondria, where it can impair complex I activity of the electron transport chain, increasing reactive oxygen species (ROS) production and mitochondrial damage [11]. Furthermore, α-synuclein drives inflammatory processes [12], not only in the substantia nigra and other regions of the brain, but possibly also in the periphery, such as in the enteric system (Fig. 1) [13, 14]. Importantly, cause-effect relationships between alterations of the peripheral and central immune system and α-synuclein pathology as well as neurodegeneration in the brain remain largely unclear to date. Furthermore, the process of aging is typically associated with alterations of peripheral immune responses and at the same time represents one of the major risk factors for neurodegenerative diseases. In this context, it remains a matter of debate to what extent these aging-dependent alterations of peripheral immune responses directly contribute to neurodegeneration in the brain, e.g., by hyper-activating immune and inflammatory pathways that may promote neurodegeneration [15].

Braak’s hypothesis suggests that α-synuclein aggregation originates in the peripheral nervous system, specifically in the anterior olfactory structures and potentially the enteric nervous system, before spreading (possibly via the vagus nerve) to the central nervous system (CNS) [16]. While initiation of pathology in the olfactory pathways and the enteric system could explain the often preceding olfactory dysfunction and chronic constipation, the Braak hypothesis has been criticized repeatedly for its validity [17]. An extension to Braak’s hypothesis is the dichotomization of PD into two subtypes: a brain-first (top-down) type, in which α-synuclein pathology starts in the brain with consecutive spreading to the peripheral autonomic nervous system; and a body-first (bottom-up) type, in which the pathology starts in the peripheral autonomic nervous system and then spreads to the brain [18]. Follow-up studies examining these two putative subtypes more closely described distinct profiles of the gut microbiome in the body-first compared to the brain-first subgroup and healthy controls [19]. Moreover, profiles of peripheral mononuclear cells (PBMC) differed between subgroups and revealed an association of monocytes with olfactory impairment, and of natural killer cells with constipation suggesting differential activation of immune cells between the two subgroups [20]. However, the body-brain hypothesis has equally been criticized and questioned for its validity [21]. Thus, while this model is intriguing, more research is necessary to confirm or refute a body- vs brain-first dichotomization of PD.

Importantly, when PD is diagnosed, more than 50% of the dopaminergic neurons in the substantia nigra have already been lost and disease pathology is quite extended [22]. Currently, available treatments for PD are symptomatic only and predominantly rely on dopamine replacement strategies [15, 23]. The development of effective curative or preventive treatments has been hampered by our inability to diagnose PD earlier in the disease process and our incomplete understanding of its pathophysiology. In this context, a more comprehensive understanding of the role of the immune system in PD pathogenesis may pave the way for effective novel strategies aimed at modulating neuroinflammatory processes early in the disease course.

In light of the critical role of the immune system in PD pathogenesis and potential treatment, the aim of this review is to provide an overview of the research methodologies currently available and frequently utilized in investigating the role of the peripheral and central immune system in PD, as well as their main findings. This review comprises state-of-the-art methodologies on genome-wide genotype data, neuroinflammation in the CNS, the impact of blood-brain barrier (BBB) disruption and dysfunction of the innate and the adaptive immune system response in the periphery, mitochondrial dysfunction and the role of infections in PD. It should be noted that this review is not a systematic review. The studies included were chosen based on the authors' expertise and experience in the field, which limits the comprehensive nature of the review. Therefore, the findings presented should be interpreted considering this limitation. Table 1 provides an overview of the methods presented in this review. We would like to note that since each topic could warrant its own review, this review provides a comprehensive but not exhaustive overview of immunological methodologies and findings in PD.

## 2. Genome-wide association analyses to study the immune system in PD

The relevance of the immune system in the etiology of PD (and other neurodegenerative diseases) can be investigated via conducting genome-wide association studies (GWAS) of idiopathic PD and related phenotypes. Approximately 5-10% of all patients suffer from a monogenic form of PD, which is caused by highly penetrant rare mutations [24, 25]. In this context, among all monogenic disease forms, homozygous loss-of function mutations in the genes *PRKN* (a.k.a. *Parkin*), *PINK1*, and *PARK7* (a.k.a. *DJ-1*) are responsible for a subset of early-onset PD, by negatively impacting mitochondrial function. Mutations have been shown to disrupt mitochondrial quality control and function. PINK1 and parkin regulate mitophagy, a key process for clearing damaged mitochondria.

**Table 1:** Overview of immune system-related technologies applied in human research in Parkinson’s disease.

Target	Scientific goals	Methods
Genome	- Identify immunologically relevant genes and variants associated with PD phenotypes	Genome-wide Association Study (GWAS)
	- **Understand biological functions, identify immune pathways relevant for PD phenotypes, identify overlaps with other immune traits, make causal inferences**	Post-hoc GWAS analyses, e.g., gene enrichment, pathway, LD Score Regression, Mendelian Randomization analyses
**PBMCs**	- Isolate PBMCs from blood for further downstream analyses	Gradient centrifugation with Ficoll-Paque medium
PBMCs/immune cell subsets	- Characterize living immune cells, e.g. -Quantify differential immune cell proportions in PD cases vs controls or across PD phenotypes -Characterize cells based on surface markers -Quantify cell surface proteins such as dopamine receptors -Quantify proteins such as cytokines/transcription factors intracellularly -Measure mitochondrial functional changes in live cells (see below) -Sort cells for further downstream analyses	Flow cytometry incl. fluorescence-activated cell sorting (FACS)/magnetic cell sorting (MACS)
	- **Quantify chemokines and cytokines and other cell markers in high resolution and with large range of markers (up to 100) in living cells**	Mass cytometry
	- **Quantify immunologically relevant gene expression (targeted), e.g., transcription factors, cytokines, dopamine receptors; quantify copy numbers of mDNA (see below)**	Real-time PCR
	- **Quantify immunologically relevant gene expression (transcriptome-wide), either in cell clusters or in single cells**	RNA sequencing (RNA-Seq)/single-cell RNA-Seq
	- **Assess immunosenescence**	Testing aging markers, e.g., telomere length, *TERT* and cyclin dependent kinase inhibitors expression
Immune cell subsets	- Evoke differential immune cell responses in activated immune cells	Cell stimulation (e.g. PHA, IL-2, anti-CD3, anti-CD28 for T cells, IFN-γ, LPS, α-syn for monocytes)
	- **Assay immune cell migration**	Transwell migration assays (e.g., SDF-1α-mediated T cell transendothelial migration) and confocal microscopy
**Immune cell subsets with phagocytosis activity**	- Assess phagocytosis activity	Bead phagocytosis assay
Fluid samples (supernatant, serum/plasma, cerebrospinal fluid)	- Quantify immunologically relevant peptides/proteins, e.g. cytokines, in liquid samples (targeted)	Enzyme-linked immunosorbent assay (ELISA); electro-chemiluminescence immunoassay (high sensitivity)
	- **Quantify immunologically relevant peptides/proteins, e.g. cytokines, in liquid samples (several thousand proteins)**	Affinity-based proteomic platforms (Olink, SomaScan) or unbiased Mass Spectrometry
Blood-brain barrier	- Localize and quantify blood-brain barrier disruption in the living human brain	Dynamic contrast-enhanced MRI
	- **Localize and quantify blood-brain barrier disruption in the post-mortem brain (quantify extravasated erythrocytes and serum proteins)**	Immunohistochemistry, immunofluorescent microscopy
Mitochondria in immune cells	- Investigate changes in mitochondrial respiration (oxygen consumption by mitochondria) including response to substrates and ADP (oxidative phosphorylation), uncoupling from ATP synthesis (LEAK), residual oxygen capacity (Rox), extracellular acidification rate	Closed chamber respirometry or microplate fluorescence-based system
	- **Investigate mitochondrial physiology states and oxidative phosphorylation/glycolysis**	Cell permeabilization, mitochondria purification and combining different respiration substrates and inhibitors
	- **Quantify mitochondrial functional changes in live cells including mitochondrial mass, mitochondrial membrane potential (MMP), ROS production**	Flow cytometry, spectrofluorometry, or fluorescence microscopy (with MMP-independent/sensitive markers, markers for mitochondria ROS)
mDNA	- Quantify mDNA copy number (mDNAcn)	qPCR, long extension PCR, digital PCR, whole-genome sequencing
	- **Quantify methylation state of mDNA**	Bisulfite sequencing; pyrosequencing; methylation-specific PCR; qPCR with methylation-sensitive and insensitive restriction enzymes; methylated DNA immunoprecipitation
	- **Investigate mDNA damage**	Long extension PCR; southern blotting; mDNA sequencing
Infection	- Assess evidence for association of infections with PD onset and progression	Epidemiological studies (e.g., cohort studies)
	- **Identify inflammatory processes or infection-related structural changes in the living human brain**	Magnetic resonance imaging (MRI), positron emission tomography
	- **Detect pathogens in blood, cerebrospinal fluid, post-mortem brain, or the gut**	qPCR; ELISA
	- **Investigate the interactions of pathogens, immune cells and neurons**	Cell culture
Post-mortem brain	- Detect, localize and quantify specific antigens (proteins, peptides) within post-mortem brains, identify cells, assess protein-protein colocalizations	Immunostaining (immunohistochemistry, immunofluorescence, immunoelectron microscopy)
	- **Detect and quantify antigens (proteins, peptides) in homogenized lysates of post-mortem brain tissue**	Immunoassay techniques (Western blotting; ELISA)
	- **Quantify gene expression in bulk tissue or in single cells**	RNA-Seq

Legend. This table presents the most established scientific methods to identify and characterize immune responses in Parkinson’s disease (PD) pathophysiology and progression. LD = linkage disequilibrium, PBMC = peripheral mononuclear cell, mDNA = mitochondrial DNA, PCR = polymerase chain reaction, TERT = telomerase reverse transcriptase, PHA = phytohemagglutinin, IL-2 = interleukin-2, CD = cluster of differentiation, IFN = interferon, LPS = Lipopolysaccharides, α-syn = α-synuclein, SDF = stromal cell-derived factor, MRI = magnetic resonance imaging, ADP = adenosine diphosphate, ATP = adenosine triphosphate, ROS = reactive oxygen species, qPCR = quantitative PCR

Dysfunction in this pathway leads to oxidative stress, which promotes α-synuclein aggregation [26]. However, the vast majority of PD (idiopathic or multifactorial PD) cases result from the combined action of dozens to hundreds of common DNA sequence variants. Individually, these variants exert only moderate to weak effects. When combined, alongside environmental and other non-genetic factors, they collectively contribute to the development of the disease [24, 25]. Genetic risk variants of multifactorial PD are most powerfully identified by GWAS. This type of study aims to identify associations between genetic variants and particular traits or diseases as outcomes on a genome-wide scale (Table 1; for details on GWAS methodology see ref. [27]). The latest and largest PD GWAS performed to date (in 49,049 PD cases, 18,785 proxy cases, i.e., individuals who do not have PD but have an affected first degree relative), and 2,458,063 control individuals of European, East Asian, Latin American and African ancestry) identified 78 genetic risk loci showing genome-wide significant (p<5x10^-8^) evidence for association with PD [28]. Once even larger datasets combined with novel molecular technologies and/or statistical methods are applied, this number is likely to increase even further. Among the 75 currently known PD risk variants many (incl. *SNCA* and *LRRK2* which also cause monogenic PD) appear to exert direct or indirect effects on immunological functions. A prominent example relates to genetic PD risk variants in the human leukocyte antigen class II (*HLA class II*) region (*HLA-DRB5*). While the exact mechanisms underlying this association is still unknown it is noteworthy that the same variants also act as *cis* expression quantitative trait loci (*cis* eQTL), meaning that they regulate the expression of *HLA class II* genes [28]. Furthermore, it has been shown that the HLA PD risk alleles present fragments of SNCA protein as antigenic epitopes and thereby drive helper and cytotoxic T cell responses in PD patients [29]. Based on the two most recent PD GWAS [28, 30], other PD risk loci with a possible effect on immunological functions include *FCGR2A*, *RAB29*, *MAP4K4*, *BST1*, *TRIM40*, *FAM49B*, *CUEDC2*, *IGSF9B*, *CD19*/*NFATC2IP*, *CYLD*, *SPPL2B, JAK1 and HS1BP3* (and there may be many more). However, it should be noted that for these and all other genetic susceptibility loci identified by GWAS, PD researchers are currently striving to understand the exact biological mechanisms underlying these statistical associations. A central challenge in this context is the extensive and complex linkage disequilibrium (i.e. correlation) structure in the human genome. This makes it exceedingly difficult to determine the functional gene(s) in a typical GWAS locus without extensive functional experiments. On a computational level, a first step towards deciphering the underlying pathophysiology are so-called post-GWAS analyses, i.e., in -depth *in silico* assessments that aim to interpret GWAS results, understand their molecular mechanisms, and prioritize findings for further, e.g., wet-lab and drug target studies (for an overview of post-hoc GWAS analyses see ref. [31, 32]). Such post-GWAS analyses have become an integral part of modern GWAS analyses and oftentimes represent the most complex and challenging task in performing this type of study. For PD, the latest GWAS yielded significant results in gene ontology set analyses suggesting the involvement of immune system pathways, specifically microglial cell proliferation, macrophage proliferation, natural killer T cell differentiation [28]. Furthermore, other recent studies using different post-GWAS analysis methods also suggested a prominent role of immune cells in PD risk. For instance, Andersen *et al.* [33] reported a significant enrichment of PD heritability in open chromatin regions of microglia and monocytes. Furthermore, a combined GWAS of PD with seven autoimmune diseases (type 1 diabetes, Crohn’s disease, ulcerative colitis, rheumatoid arthritis, celiac disease, psoriasis, and multiple sclerosis) identified 17 shared loci, suggesting a prominent role of the immune system in PD [34]. Furthermore, most recently, we assessed whether immune cell compositions are already altered in healthy individuals at genetic risk for PD as quantified by so-called polygenic risk scores (PGS) [35]. A PGS summarizes the number of GWAS-derived genetic PD risk alleles per individual weighted by their effect size estimates. While we observed some weak, nominally significant results, this study suggests that major alterations of immune cells in peripheral blood are not yet visible in healthy individuals at increased genetic risk and likely only occur later in the course of PD [35].

Taken together, these studies are excellent examples of the potential of “re-using” existing GWAS data and emphasize the importance of data sharing in the field of complex disease genetics. Gaining ready access to GWAS summary statistics and/or individual-level genotyping data provides an invaluable opportunity for PD geneticists, but also for researchers from other fields (including immunologists), to re-analyze already existing data under different hypotheses, using different analysis strategies, and integrating additional datasets. Eventually, this open-data strategy will further our understanding of PD genetics, including the role that immune system (dys)function plays in multifactorial PD.

## 3. Neuroinflammation investigated in post-mortem brains

Neuroinflammatory processes in the CNS have been recognized as key contributors to neurodegeneration and progression in PD. McGeer *et al*. [36] described for the first time an involvement of the central immune system in PD. They described numerous reactive microglia phagocytosing dopaminergic cells in the substantia nigra utilizing HLA-DR staining. Microglia are resident macrophages, i.e., innate immune cells, and the primary immune cells of the CNS. Indeed, neuroinflammation is driven and exacerbated by microglia, in conjunction with astrocytes, a glial cell subtype and the most common cell in the CNS (Fig. 1) [37, 38]. In this context, the investigation of post-mortem brains from PD patients has significantly contributed to recognizing the importance of the neuroinflammatory cascade. The most frequently employed methods comprise immunostaining (such as immunehistochemistry, immunofluorescence, and immuneelectron microscopy) as well as Western blotting (immunoblotting) and the enzyme-linked immunosorbent assay (ELISA) technique. Specifically, microglia become activated by and internalize misfolded or aggregated α-synuclein [39] (Fig. 1). This process is originally a compensatory response to clear toxic protein aggregates, protect neurons and maintain homeostasis. However, in PD, excessive or prolonged exposure to α-synuclein, e.g., released by neurons, can lead to chronic activation of microglia. Furthermore, mitochondrial dysfunction in PD likely plays a role in triggering microglia activation and neuroinflammation. There is some evidence that mitochondrial dysfunction may occur before neuroinflammation: Specifically, mitochondrial damage results in the release of “mitochondrial-derived damage-associated molecular patterns” (DAMP) that are recognized as danger signals and trigger inflammatory responses (for review see e.g., ref. [40]). Activation of microglia results in the continuous release of pro-inflammatory cytokines (TNF-α, IL-1β, IL-6) [41], reactive oxygen species (ROS), and nitric oxide (NO), resulting in even more oxidative stress and neuronal damage [42-44]. Interestingly, a recent study showed that human dopaminergic neurons (derived from induced pluripotent stem cells) develop Lewy body-like inclusions only when treated with both α-synuclein preformed fibrils and a proinflammatory cytokine (interferon-γ or IL-1β) or when co-cultured with activated microglia-like cells [45]. This may indicate that the presence of inflammatory stimuli is essential for the development of Lewy body pathology. Studies applying immunostaining techniques in human brain described abnormal accumulation of non-amyloid-β component precursor (NACP)/α-synuclein in microglia in PD [46, 47]. Surprisingly, a recent study - in mice - suggested that α-synuclein accumulation in microglia but not neurons may drive dopaminergic neuron degeneration. Microglia activated by α-synuclein overexpression showed phagocytic exhaustion and produced oxidative and proinflammatory molecules that provoked peripheral immune cells to migrate into the CNS even in the absence of α-synuclein aggregation in dopaminergic neurons [48]. However, these findings need to be substantiated by further investigations in human cells. Furthermore, in the human brain, immunostaining showed the presence of ICAM-1-positive reactive astrocytes as a sign of sustained neuroinflammation [49]. Indeed, data suggest that besides microglia, astrocytes also play a central role in neuroinflammation: in affected regions in post-mortem PD brains, reactive astrocytes that can also uptake and become activated by α-synuclein were reported with a pro-inflammatory and neurotoxic phenotype (for review see [50]). Liddlelow *et al*. [51] showed that activated microglia induce neurotoxic reactive astrocytes, i.e., glial fibrillary acidic protein (GFAP)- and S100 calcium-binding protein B (S100β)-positive A1 astrocytes with high expression of complement component C3, which are abundant in neurodegenerative diseases. Furthermore, astrocytes appear to contribute to the spreading of toxic α-synuclein aggregates and subsequently neuroinflammation by intercellular transfer of α-synuclein via so-called tunneling nanotubes [52]. Such tunneling nanotubes have been described for microglia as well [53]. The involvement of microglial pro-inflammatory signaling in PD pathogenesis has been validated in recent studies that emphasized a role of the microglial NOD-like receptor family pyrin domain-containing 3 (NLRP3) inflammasome in PD [54-59] (Fig. 1). The NLRP3 inflammasome facilitates caspase-1 activation and the release of proinflammatory cytokines IL-1β/IL-18 in response to infection, sterile inflammation, or cellular damage associated with neurodegeneration [60, 61]. Gordon *et al*. [54] demonstrated increased inflammasome activation at the sites of dopaminergic cell loss in patients with end-stage PD by quantifying cleaved caspase-1 and inflammasome adaptor protein (ASC) in brain lysates of PD patients and controls by Western blotting. Immunohistochemistry confirmed NLRP3 and ASC upregulation in PD substantia nigra, predominately in microglia. A more recent addition to the techniques used on human post-mortem brains is bulk or single-cell RNA sequencing on a transcriptome-wide scale and other omics studies. For instance, a pilot study on >41,000 single-nuclei transcriptomes of post-mortem midbrain from six idiopathic PD patients and five controls supported the previous finding (see above) of glial activation as a central mechanism in the pathology of PD [62].

The compromise of astrocyte function may contribute to a dysfunction of the BBB as astrocyte end-feet are an integral part of the BBB (see below). A dysfunctional BBB is a prerequisite for the involvement of peripheral adaptive immune cells migrating into the CNS. Along these lines, besides HLA-DR+ microglia the above mentioned study by McGeer *et al*. [36] also already described CD3+ T cells in post-mortem brains of PD patients. Using immunohistochemistry, both CD4+ and CD8+ T cells but not B cells have been described in post-mortem PD brains [63]. In this context, astrocytes can act as antigen-presenting cells, and MHC-II-expressing astrocytes were found in proximity to CD4^+^ T cells in post-mortem PD brain tissue. Sulzer et al. showed that T cells recognize epitopes derived from α-synuclein. This may indicate that astrocytes activate T cells in the PD brain [52] (Fig. 1). Comparing diseased patients and controls, α-synuclein specific T cell responses were highest shortly after diagnosis of PD and then decreased [64]. Interestingly, Galiano-Landeira *et al*. [65] performed an immunohisto-chemistry/immune-fluorescence quantitative and phenotypic assessment of T cells infiltrating in human post-mortem substantia nigra and correlated these with neuronal death and synucleinopathy throughout different stages of the disease. They showed that CD8+ T cells (but not CD4+ cells) were increased in PD cases compared to controls already in early stage of the disease, when substantia nigra α-synuclein aggregation and dopaminergic neuronal death was yet absent. Central CD4+ and CD8+ cells may escalate the neuroinflammatory situation even further: CD4^+^ lymphocytes secrete pro-inflammatory cytokines (IFN-γ, TNF-α, and IL-17), which stimulate microglia to produce high levels of neurotoxic factors, including ROS, glutamate, and TNF-α. Furthermore, it has been suggested that MHC-I expression in neurons can be upregulated by microglia suggesting a possibly direct neurotoxic effect of cytotoxic CD8+ cells [66] (Fig. 1).

## 4. Neuroinflammation investigated by PET imaging

PET imaging of the brain can target specific immune cells, proteins such as α-synuclein, or specific disease processes with the potential to improve diagnosis, stratify patients, monitor disease progression and eventually develop improved or novel treatments. Examples of clinically relevant methodologies for diagnostic purposes are dopaminergic imaging and cardiac meta-iodobenzylguanidine (MIBG) scintigraphy (reviewed in ref. [67]). In this context, PET imaging has also been applied to visualize neuroinflammation in PD by targeting immune components. Most frequently this technique aims to target activated microglia [67]. Specifically, the most commonly used radioligand 11C(R)PK11195 binds to the mitochondrial translator protein TSPO expressed in activated microglia. This ligand has been reported to show increased binding in cortical regions in PD and to show inverse correlation with cognition in PD [68]. However, results remain inconclusive: Other studies have reported increased binding in the substantia nigra and putamen but not the cortex [69] or to correlate with preserved cognition and white matter integrity in PD [70]. Importantly, using a multi-faceted approach a recent study demonstrated that microglial TSPO expression increases with pro-inflammatory stimuli or in neurodegenerative disease settings in rodent models but not in humans [67, 71]. Since this study comprehensively shows that the TSPO ligand signal may not reflect microglial activation in humans, a careful interpretation of PET studies using 11C(R)PK11195 is warranted, and new targets appear to be necessary to assess the role of microglia activation in the living brain in PD. In this context, post-mortem autoradiography, a technique used to visualize the distribution of radiolabelled molecules in tissues after death, may be instrumental. Before clinical use, new PET tracers can be tested in animal models or post-mortem human tissues using autoradiography to assess affinity, specificity, and regional binding patterns. This technique has also been successfully applied to evaluate other neurodegenerative markers such as tau PET tracers (e.g. ref. [72]). It provides high spatial resolution, quantification, and off-target assessment and thus helps to refine the design and selection of novel PET radioligands.

## 5. The blood-brain barrier in PD

The blood vessels, specifically the capillaries, that vascularize the brain have unique properties, which allow them to tightly regulate the passage of ions, small molecules (including toxins) and blood cells between the blood and the brain. This BBB is formed by endothelial cells of the capillaries, astrocyte end-feet ensheathing the capillaries, and pericytes located in the capillary basement membrane. The BBB maintains CNS homeostasis, allows for proper neuronal function, and protects the CNS from toxins, pathogens, and inflammation. However, different conditions such as aging, neurodegenerative processes including neuroinflammation as well as systemic inflammation can affect the integrity of the BBB and allow immune and other cells and particles to enter the CNS [73-76] (Fig. 1).

In PD patients, BBB disruption and impaired functioning have been documented via imaging studies [77], single cell transcriptomics [78], CSF angiogenesis markers [79] and postmortem brain tissue evaluations [80]: For instance, using dynamic contrast enhanced magnetic resonance imaging, Al-Bachari *et al*. [77] reported on subtle BBB disruptions in PD in the substantia nigra, white matter and posterior cortical regions. Furthermore, Gray & Woulfe showed compromised BBB integrity in post-mortem striatum samples of PD patients *vs* unaffected controls including erythrocyte extravasation, perivascular hemosiderin, and leakage of various serum proteins into brain parenchyma by immunohistochemistry and immunofluorescent microscopy [80]. In addition, based on single-cell RNA sequencing of peripheral T cells, Yan *et al*. [78] postulated that peripheral cytotoxic CD4+ T cells influence BBB function by migrating to mesencephalic endothelial cells and activating the IFN-γ response in these cells [78]. Janelidze *et al*. [79] described significant associations of CSF levels of angiogenesis markers (VEGF, VEGF receptors, placental growth factor; angiopoietin 2, and IL-8) in PD patients compared to controls. Increased levels of angiogenesis markers were correlated with BBB disruption in imaging.

## 6. Peripheral immune response in PD

While neuroinflammation certainly plays a central role in PD pathogenesis and progression, many studies have shown peripheral immune system changes in PD as well (Fig. 1) [81, 82]. It has been postulated that inflammation may start in the periphery, at least in a subset of PD patients (concordant with the body-first hypothesis, see above) and that activated immune cells then migrate through a disrupted BBB (see above) in the CNS [83]. In general, the current knowledge about the role of the peripheral immune system remains incomplete with conflicting results across studies, which may be due to heterogeneous selection of cases and controls but also methodological differences (see Conclusion section). To study specific immune cell subsets, which accomplish different immunological functions, relevant cells can be isolated from PBMCs using techniques like flow cytometry, including magnetic cell sorting (MACS) and fluorescence-activated cell sorting (FACS). Flow cytometry enables rapid analysis of numerous cells simultaneously, primarily focusing on surface markers [84]. However, it can also be used to measure cytokine production intracellularly (Table 1; for details on flow cytometry methodology see ref. [85]). Other analytical techniques commonly used are real-time PCR (RT-PCR) and RNA sequencing (RNA-Seq), including single-cell RNA-Seq (Table 1). Mass cytometry can also assess chemokines' and cytokines' expression in specific cell clusters [86] (Table 1, for details on mass cytometry see ref. [87]).

### 6.1 Adaptive immune cells

The published literature focused predominately on adaptive immune cells, particularly on the role of T lymphocytes. Based on the cellular surface proteins they express, T cells can be categorized into CD4+ T cells (a.k.a. T helper cells [Th]), and CD8+ T cells (a.k.a. cytotoxic T cells). In general, lower numbers have been reported repeatedly for CD4+ T cells as well as CD8+ cells in PD [88] (Fig. 1).

#### CD4+ T cells

CD4+ T cells have been described as critical players in both the pathophysiology [81, 89] and clinical and diagnostic aspects of PD [90, 91]. These cells include several subsets with distinct immunological roles, such as Th1, Th2, Th17, and regulatory T cells (Tregs). A range of studies employed flow cytometry to quantify and compare peripheral CD4+ T cell subtypes in PD, not only comparing healthy subjects, drug-treated and/or drug-naive PD patients (e.g. ref. [81]), but also analyzing immune cell subtypes patients at different disease stages (e.g. ref. [92, 93]) and with disease phenotypes (e.g., [90, 91, 94]. However, especially for disease progression and phenotypes, studies have reported differing results, which is likely due to a combination of small sample sizes, heterogeneous study populations, and methodological differences. Specifically, i) Th1 cells primarily participate in immune responses against intracellular pathogens such as mycobacterial species and viruses. [95]. In PD, the number and frequency of Th1 cells seem to be increased, contributing to elevated tumor necrosis factor α (TNF-α) and interferon γ (IFN-γ) plasma levels, which are key mediators of neuroinflammation [81, 96] (Fig. 1). This increase in Th1 cells was described in both drug-naïve patients and in patients on dopaminergic drugs, suggesting that antiparkinson drugs do not confound this observation [81]. ii) Th2 cells participate in immune responses against large extracellular pathogens such as helminths, expressing cytokines like interleukin (IL)-4, IL-5, IL-13, and the transcription factor GATA3 [95]. In PD, few studies have reported on reductions in the absolute number and frequency of Th2 cells in PD patients vs controls (Fig. 1); however, results have been inconclusive and partially contradictory [81, 89, 93]. iii) Th17 cells primarily participate in immune responses against extracellular pathogens while they play a central role in the pathophysiology of a number of autoimmune diseases [95]. In a meta-analysis, they showed an increase in peripheral blood of PD patients versus controls (Fig. 1) and a positive association with motor dysfunction in PD but there was substantial heterogeneity across studies [97]. iv) Tregs play a crucial role in preserving immunological tolerance and maintaining immune homeostasis. They express regulatory cytokines such as IL-10, TGFβ, and IL-3, and the transcription factor Foxp3 [95]. In the context of PD, Tregs may help regulate excessive immune responses and inflammation, potentially modifying disease progression. However, findings in human PD have been inconclusive with some studies reporting a decrease (e.g. ref. [81, 89, 98-100]) while others have not found any changes [101-103]).

When analyzing the immune profiles of PD patients, it is relevant to not only investigate immune cell frequencies but also to evaluate their functionalities such as cytokine production, expression levels of transcription factors or of dopamine receptors, and assessment of mitochondrial function. To this end, CD4+ T cell subsets can be examined using different experimental conditions: cells can be cultured in resting state or using stimulating agents; the latter mimics the changes occurring during an *in vivo* inflammatory condition [104, 105]. For example, studies using activating agents like IL-2/PHA and anti-CD3/anti-CD28 have shown in CD4+ T cells a dysregulation of dopamine receptor expression [81, 105, 106]: Kustrimovic *et al.*, through flow-cytometry analysis of cell phenotype found a significant reduction in D1-like receptors (DRD1 and DRD5) on naïve cells CD4+ T cells and an increase in D2-like receptors (DRD3 and DRD4) on effector memory CD4+ T cells [107], which was positively associated with worse motor symptomatology [107]. Conversely, Elgueta *et al.* reported a reduced DRD3 in ex vivo activated CD4+ T cells of patients, and this downregulation correlated with disease activity measured by UPDRS [105]; however, it seems that this profile is related to cell activation and not present in basal conditions. Interestingly, Kustrimovic *et al.* [107] did not find a differential expression of *DRD3* in resting CD4+ T cells of PD patients vs controls when using flow cytometry, although they found different levels of *DRD3* mRNAs by RT-PCR. This underlines the importance of performing analyses under different conditions (e.g., activated and resting state) and of validating results using different techniques such as flow cytometry and RT-PCR [78].

To assess immune dysregulation in PD, cytokines can be informative as they play a central role in regulating neuroinflammatory processes. Numerous studies measured plasma or serum cytokines [93, 98, 99]. However, in these settings it is not possible to determine the exact immune cell types that released the cytokines into the blood fluid. It is worth noting that most cytokines can be produced by many different cell types [108], and to our knowledge, only few studies assessed cytokines produced by specific cell types. In this context, the two main methods to quantify cytokines are flow cytometry (with the need of blocking cytokine secretion prior to quantification) or ELISA to quantify cytokine production in the supernatant (Table 1). Therefore, we will focus exclusively on studies that measure cytokine production in a cell-type-specific manner. The quantification of cytokine production by a specific cell population is primarily achieved through flow cytometry and ELISA techniques. Results have been seemingly contradictory: Cook *et al*. through a flow cytometric evaluation, found no differences in IFN-γ, TNF-α, and IL-2 production in CD4+ T cells between healthy individuals and patients after 18 hours of stimulation [109]. Similarly, with a shorter stimulation time of 4 hours, Yan Z. *et al*. reported no differences in intracellular IFN-γ production in Th1 cells. However, they observed increased production of IL-17A in Th17 cells and IL-4 in Th2 cells, as determined by flow cytometry [93]. In contrast, Kustrimovic *et al*., using ELISA to analyze supernatants from activated CD4+ T cells after 48 hours, found significantly elevated production of IFN-γ and TNF-α in T effector cells from both drug-naïve and treated PD patients compared to healthy controls. Meanwhile, levels of IL-4, IL-17A, and IL-10 remained unaffected in T effector cells from patients [81]. Similarly, Mamula *et al*. observed increased levels of IFN-γ in supernatants from activated CD4+ T cells in patients after 48 hours of stimulation [106]. Such discrepancies are likely due to studying different immune cell populations, using different stimulation times and methodology applied and including heterogeneous patient populations.

Similarly to quantifying cytokines, investigating transcription factors may reveal regulatory mechanisms of gene expression implicated in PD. Along these lines, transcription factors in PD were quantified by flow cytometry [105] or by RT-PCR in CD4+ T cells [81, 90, 110]. For instance, Kustrimovic *et al*. [81] showed that PD patients had lower levels of *TBX21*, *STAT3*, *STAT4* and *NR4A2* and higher levels of *STAT6*, *GATA3*, and *FOXP3* when compared to healthy controls. The expression of *TBX21*, a key transcription factor of Th1 differentiation, was increased in both PD and idiopathic rapid eye movement sleep behaviour disorder patients, a known prodromal symptom of PD, suggesting early involvement of the immune system [111].

Multiple other functional aspects of CD4+ cells can be examined. For instance, Mamula *et al*. [106] showed altered migration potentials of CD4+ T cells in PD patients vs controls using a multifaceted approach including transwell migration assays, as well as impaired mitochondrial positioning within the cell and reduced mitochondrial functionality. In addition, expression levels of *S100A10* (a.k.a. *P11*) in CD4+ Th1, Th2 and Th17 subsets in PD patients were increased compared to controls [106].

#### CD8+ T cells

Similar to CD4+ T cells, CD8+ T cells comprise diverse subsets of cells that play different roles in immune regulation and cytotoxicity: These subsets include naïve, effector, memory, and tissue-resident memory CD8+ T cells, as well as regulatory CD8+ T cells (CD8+ Tregs). CD8+ T cells are well known for their cytotoxic functions, and they are also involved in neurodegeneration through the release of pro-inflammatory cytokines and direct neuronal damage [65, 93]. Notably, two human post-mortem studies have shown CD8+ T cell infiltrations in the brains of PD patients suggesting a direct link between peripheral immune activation and CNS pathology [63, 65]. Similar to the studies on CD4+ cells, flow cytometry is a popular methodology to characterize CD8+ cells [89, 92, 94, 99, 103, 112-116] including their specific subsets and differentiation stages [93, 98, 117-119]. Flow cytometry has also been employed to investigate intracellular cytokine production in CD8+ T cells [93]. Several studies have reported a range of differential frequencies and characteristics of CD8+ T subsets in PD patients vs controls such as decreased replicative senescence (a marker of normal aging) in total CD8+ T cells [119], a decrease in naive CD8+ T cells [93], an increase in IFN-γ-producing CD8+ T cells [93], an increase in terminally-differentiated effector memory (TEMRA) CD8+ T cells and CD8+ NK T cells (Fig. 1) and a decrease in CD8+FOXP3+ T cells [118], as well as a decrease in IL-10-producing CD8+ Tregs [98]. CD8 T-cells derived from PD patients show a profile suggesting a lack of immunosenescence, i.e., a lack of aging effects in immune cells. For example, the number of CD8+ TEMRA cells was found to be significantly reduced in patients, which could make them more prone to a reactive pro-inflammatory response [92, 94, 115]. Furthermore, Kouli *et al.* [94] assessed immunosenescence in CD8+ T cells in PD patients vs controls by testing several aging markers such as telomere length, the expression levels of the telomerase reverse transcriptase enzyme (*TERT)* and of cyclin dependent kinase inhibitors (p16^INK4a^ and p21^CIP1/Waf1^). They observed a reduction in CD8+ T cell replicative senescence.

### 6.2 Innate immune cells

The innate immune response serves as the body's first line of defense against microbial pathogens, can initiate adaptive immune responses, and plays a role in tissue repair [120, 121]. Myeloid cells are the main cellular component of innate immune responses. They include monocytes and macrophages, granulocytes (i.e., neutrophils, basophils, and eosinophils), and dendritic cells (DC) [122]. Additionally, microglia represent the major class of myeloid cells in the CNS (see above) [123]. As monocytes and DCs have been the focus of numerous studies investigating the role of the peripheral innate immune system in PD, the following section will provide an in-depth review of the published research on these cell types in PD (Fig. 1).

#### Monocytes

Recent studies suggested that monocytes may serve as contributing factors to PD pathogenesis. They are effector cells of the innate immune system and patrol the bloodstream, phagocytose debris, communicate with local cells, and give rise to macrophages or DCs [124-126].

While some studies reported increased circulating classical (CD14+CD16-) and decreased intermediate (CD14+CD16+) and non-classical monocyte frequencies (CD14-CD16+) in PD, others failed to find statistically significant differences in PD [126-129]. These seemingly contradictory results may - at least in part - originate from different methodological approaches across studies: For instance, Grozdanov *et al.* [127] used a human primary monocyte culture system and FACS on whole blood to determine the state of monocytes from PD patients during the disease course. This approach allowed the authors to differentiate monocytes from other leukocytes and further distinguish classical (CD14+CD16-) from non-classical (CD14-CD16+) monocytes. The authors also included the intermediate population (CD14+CD16+) in the non-classical group, referring to this mixed cell group as the CD16+ population following the nomenclature proposed by [130]. The authors demonstrated an enrichment of classical monocytes and a reduction in CD16+ monocytes in the peripheral blood of PD patients compared to healthy controls. A similar observation was reported by Wijeyekoon *et al*. [129] after isolating CD14+ cells from PBMCs using MACS and characterizing them using flow cytometry. However, other studies have reported contradictory findings. For instance, Schlachetzki *et al*. [126] utilized monocytes isolated from PBMCs by negative selection with magnetic beads. These monocytes were then either immediately subjected to FACS or pelleted, lysed, and stored at -80°C until RNA isolation. Subsequent flow cytometry analysis based on CD14 and CD16 expression revealed no significant differences in the distribution of classical (CD14+CD16-), intermediate (CD14+CD16+), and non-classical (CD14dimCD16+; CD14dim denotes low amounts of CD14) monocyte subpopulations between PD patients and controls [126]. Furthermore, in a study performed on peripheral blood and CSF, subpopulation analysis of innate immune cells using immune cell profiling by multiparameter FACS [128] revealed a shift in cell proportions from classical monocytes (defined as CD14+CD16-) to non-classical monocytes (CD14+CD16+) in the CSF of PD vs control individuals, but no differences in peripheral blood. It is noteworthy that the two latter studies [126, 128] did not include the intermediate population (CD14+CD16+) in the non-classical population. Another study reported on observed sex-specific differential gene expression in monocytes of PD vs control individuals and showed an inflammatory activation of monocytes in females with PD with an enrichment of gene sets associated with IFN-γ stimulation [131].

Cellular responses of monocytes have been investigated by PD researchers by several techniques that used cell cultures in the presence of a range of different activating stimuli (e.g., LPS and α-syn) at different concentrations and incubation times. These methodological differences have likely contributed to contradictory results: For example, Grozdanov *et al.* [127] allowed monocytes to rest for 24h prior to LPS stimulation (1ng/ml, 24h-incubation). The authors observed increased sensitivity to LPS in monocytes of PD patients compared to controls [127] using cytokine measurements (by ELISA and by electro-chemiluminescence immunoassay [which is comparable to an ELISA with different capture surfaces and readouts]) and bead phagocytosis assay. Nissen *et al*. [132] used thawed PBMCs and observed decreased sensitivity to LPS (100ng/ml, 1h-incubation) and fibrillar α-syn (100ng/ml, 24h-incubation) when quantifying CD163 and cytokines by ELISA. Finally, Wijeyekoon *et al.* [133] did not observe any differences in response to LPS (1ng/ml, 24h-incubation) when measuring cytokine secretion using an electrochemiluminescence assay.

#### Dendritic cells

DCs provide a key link between the innate and adaptive immune systems. They initiate and regulate pathogen-specific adaptive immune responses and are central to the development of immunological memory and tolerance [134]. As DCs are important players of the brain immune surveillance, the investigation of subsets infiltrated into brain tissue would reveal important insights into PD pathogenesis and progression, but this is particularly challenging due to the restricted number of DCs in the brain, morphological heterogeneity, and lack of specific cellular markers [135, 136]. Thus, most studies focused on circulating DCs in peripheral blood using FACS and - unlike the studies on monocytes presented above - mainly showed consistent findings. These studies predominately investigated the two main subpopulations of blood DCs that are classified according to their surface markers, namely conventional or classical (cDCs; lin- CD11c+ HLD-DR+, CD123lo; a.k.a. myeloid DCs) and plasmacytoid DCs (pDCs; lin- CD11c- HLD-DR+, CD123hi) [135]: cDCs can influence different types of responses, from tolerance/regulation (e.g., induction of Tregs) to innate and/or adaptive (Th1, Th2, Th17, T follicular helper cells, cytotoxic T lymphocytes) immune responses [137] pDCs are known for their capacity to produce type I interferons upon infection. These cells primarily originate from lymphoid precursors and are ontogenetically distinct from dendritic cells [137].

Importantly, using FACS on fresh whole flood, PD patients were reported to show lower levels of circulating DCs (mainly the conventional subset) compared to controls (Fig. 1). Furthermore, the number of both cDCs and pDCs was inversely associated with motor symptom severity [138]. However, using FACS on PBMCs from naïve PD patients, another study [98] reported that conventional cDCs were not different in PD patients compared to controls, contrasting with the findings of Ciaramella *et al*. [138]. Interestingly, the same study [98] reported that CD11c+PD-L1+ DCs were decreased in PD patients compared to controls [98]. Notably, PD-L1 on DCs plays a critical role in limiting T cell responses [139]. More recently, [140], observed a decrease in cDCs using flow cytometry on PBMCs, which corroborates the prior reports of reduced DCs in PD patients compared to controls [98]. When interpretating the discrepant results of these studies, it should be taken into consideration that in the study of Ciaramella *et al*. [96], roughly one third of the PD patients was treated with Levodopa (L-dopa), one third was treated with dopamine agonists and one third was treated with both, while less than 10% were drug naïve. In this context, it is noteworthy that dopaminergic medication has immunomodulatory effects [141], which may have impacted the immune profiles of the investigated PD patients.

## 7. Mitochondrial homeostasis in human blood cells in PD

As outlined above, cumulative oxidative stress, disrupted mitochondrial respiration, and mitochondrial damage have been implicated in neurodegenerative diseases, including PD [142, 143]. Numerous studies showed that beyond immune and neuronal cells in the CNS, also PBMCs exhibit mitochondrial dysfunction in neurodegenerative diseases like PD and Alzheimer's disease [144, 145]. Furthermore, it is well established that the genes *PRKN*, *PINK1*, and *PARK7*, which may harbor loss-of-function mutations that cause the autosomal recessive form of monogenic PD, are regulators of mitochondrial homeostasis and quality control [146]. Besides their known roles in maintaining cellular homeostasis, recent work suggests that Pink1/Parkin-mediated mitophagy (i.e., removing defective mitochondria by autophagy) restrains innate immunity and attenuates inflammasome activation [147-149]. In this context, Prkn-/- and Pink1-/- mice showed a strong inflammatory phenotype under mitochondrial stress, and human *PINK1*/*PRKN* mutation carriers displayed elevated cytokines in the blood [148]. Similar metabolic switches were also observed in macrophages, B cells, and other immune cells during their differentiation or activation [150, 151]. However, until now, most PD studies used PBMCs (alongside platelets) to evaluate mitochondrial homeostasis in different disease conditions. Given that the bioenergetic profiles of different blood cells are distinct, cell-specific changes in mitochondrial function will need to be considered in future studies to understand the mitochondrial contribution to PD pathogenesis. This was showcased by a proof-of-concept study that used a high-throughput mitochondrial phenotyping platform to investigate multiple mitochondrial parameters in various immune cell subtypes from healthy blood donors. The study compared these findings to results obtained from mixed PMBCs. Results in PMBCs appeared to be affected by different distributions of cell subtypes, contamination with platelets, and week-to-week changes in mitochondrial activities [152]. Such in-depth analysis of mitochondrial phenotypes considering distinct blood cell subtypes has yet to be applied in PD research.

### 7.1 Mitochondrial respiration, reactive oxygen species and mitochondrial potential

Studies investigating mitochondrial respiratory activity in PBMCs of PD patients vs controls using either chamber based platinum electrodes or a microplate fluorescence-based system (Table 1) have yielded contradictory results with both increased and decreased respiration parameters [153-155]. These may, at least in part, be attributable to differences in the ascertainment of study participants, limited sample sizes, as well as differences in laboratory and analysis protocols. During mitochondrial respiration, ROS are produced from the electron transport chain, mostly from complex I and III [156]. Low amounts of ROS play a role as a signaling molecule and exhibit beneficial effects for immune cells. High levels of ROS can lead to DNA damage and apoptosis in different cell types, and also modulate immune responses such as production of inflammatory cytokines, chemotaxis, as well as macrophage and T helper cell polarization [157-161]. Mitochondrial ROS, mitochondrial membrane potential (MMP), morphology and mass can be assessed by flow cytometry, spectrofluorometry, or fluorescence microscopy-based techniques using mitochondria-specific fluorescent dyes. A few studies measured the changes in MMP in PBMCs of PD patients vs controls: for instance, Qadri *et al*. [162] measured the MMP in PBMCs of PD and healthy individuals by flow cytometry using a lipophilic cationic dye (JC-1). They found that the MMP was significantly lower in PD patients compared to healthy controls. Smith *et al.* [155] measured mitochondrial content (using MitoTracker) and mitochondrial ROS production (using MitoSox) by flow cytometry in PD patients and controls using peripheral monocytes, lymphocytes, and total PBMCs. They observed that PD patients' monocytes had considerably greater amounts of mitochondrial ROS and significantly lower mitochondrial mass compared to those of control individuals. In these studies, fresh blood samples were used to isolate PBMCs, monocytes and/or lymphocytes, and flow cytometry was employed to measure mitochondrial functional changes in live cells. Furthermore, Annesley *et al*. [163] reported that the ROS levels in immortalized lymphoblasts were significantly higher in PD than in control individuals. In fact, PD lymphoblasts showed hyperactivity with increased rates of mitochondrial respiration. In line with this, Ming *et al*. [164] showed that ROS levels measured by fluorometry were significantly enhanced in peripheral blood lymphocytes from both sporadic PD patients and those with the *PARK2* C441R mutation compared to healthy controls. When comparing PBMCs from PD patients receiving L-Dopa to those from healthy participants, Prigione *et al*. [165] found noticeably higher levels of oxidative stress, which they linked to increased ROS generation. Controversial results on the role of mitochondrial homeostasis in PBMCs in PD may be due to the use of different cell sources, such as peripheral blood cells or lymphoblastic cells. Since lymphoblastic cells are immortalized cells derived from the patients’ lymphocytes their metabolic phenotype can be different from that of peripheral blood cells.

### 7.2 Mitochondrial DNA

Given the role of mitochondria dysfunction on immune cell function and in PD, a series of studies have been performed to investigate the role of alterations of mitochondrial DNA (mtDNA), i.e., mtDNA copy numbers (mtDNA-CN), epigenetic changes and mutation rates, in various PD models as well as in patient samples. Although the corresponding methodologies have constantly improved in recent years, the results across the currently published studies are contradictory and overall largely inconclusive [166]. This may at least partly be due to sample ascertainment schemes, but also different laboratory and analytical approaches. Importantly, no highly penetrant mtDNA mutation has yet been described for PD. While somatic mtDNA point mutations and deletions have been described to be enriched in PD [167, 168], these are most likely a consequence of the cellular and mitochondrial dysfunction and damage occurring in PD.

## 8. Role of infections in PD

It has been speculated that pathogens (e.g., viruses or bacteria) may act as “triggers” for the pathophysiological processes entering via the nasal and intestinal epithelium years or even decades before the neuropathological changes in the substantia nigra occur (Fig. 1). The gut and the brain communicate bidirectionally through a complex network of neural, hormonal, and immune pathways known as the gut-brain axis. Gut microbiota play a vital role in modulating this communication, producing various metabolites, neurotransmitters, and immune molecules affecting brain function, potentially influencing the development and progression of PD [169]. It has been proposed that certain gastrointestinal infections could induce alterations in the gut microbiome, resulting in increased intestinal permeability (leaky gut) and immune activation, potentially contributing to inflammation and systemic effects impacting PD [170].

Epidemiological studies have reported that infections with neurotropic viruses such as the influenza virus, herpes simplex virus, and enteric viruses (e.g., hepatitis C virus and rotavirus) may increase the risk of developing PD [171]. Furthermore, human immunodeficiency virus (HIV) has been associated with the development of PD-like symptoms [172]. Interestingly, a few cases of incident PD or parkinsonism have been described following severe acute respiratory syndrome coronavirus 2 (SARS-CoV-2) infection. However, long-term data investigating whether SARS-CoV-2 induces PD do not yet exist [173]. While PD itself may not be associated with an increased susceptibility to the virus, individuals with PD who contract COVID-19 may experience a temporary worsening of their symptoms [174]. Furthermore, epidemiological studies have provided suggestive evidence linking a history of tuberculosis to a higher risk of developing PD [175, 176], observing an increased PD incidence among individuals with a previous tuberculosis diagnosis compared to the general population. In this context it is noteworthy that genetic variants in *LRRK2* have been reported to increase susceptibility to PD and also to mycobacterial infections [177]. However, it is crucial to acknowledge that these studies establish an association rather than a definitive cause-and-effect relationship.

*In vitro* models, specifically cell cultures, may be used to evaluate the functional impact of infections on neurons, to characterize the immune response of glial cells and to investigate the intricate interactions of pathogens, immune cells and neurons. Employing immunological analyses, including techniques such as immunofluorescence, immunoblotting, and ELISA, has been central in detecting and quantifying immunoreactive molecules, cytokines, and other inflammation-related mediators. Also, the use of animal models (which is not the topic of this review) has played a crucial role in investigating the impact of infections on the nervous system and assessing inflammation, immune responses, and potential pathological alterations in PD. Significant findings have been obtained regarding the impact of different viral and bacterial infections. For instance, infections with H1N1 influenza virus in Lund human mesencephalic dopaminergic cells *in vitro* (as well as in Rag knockout mice, i.e., mice lacking functional B and T lymphocytes) lead to the formation of α-synuclein and Disrupted-in-Schizophrenia 1 (DISC1) aggregates. Additionally, Oseltamivir phosphate, an anti-influenza drug, prevents H1N1-induced α-synuclein aggregation [178]. Moreover, a potential link between PD and *Mycobacterium avium subsp. paratuberculosis* (MAP), causing Johne's disease in animals with zoonotic potential, has garnered scientific attention: *In silico* analyses identified conserved regions shared between MAP and human α-synuclein. Furthermore, ELISA revealed a robust humoral response against MAP antigen in the sera of PD patients, and cross-reactive antibodies against mycobacterial proteins and human α-synuclein were also detected [5]. Defects in the PD genes *LRRK2* and *PRKN* may create a permissive environment for MAP infection while impairing xenophagy, the process of clearing intracellular pathogens [179, 180]. MAP, originating from an enteric infection, might initiate a pathological process through the vagus nerve, leading to targeted neuroinvasion in the CNS, as suggested by recent studies involving Listeria [181]. Interestingly, it has been shown that viral infection downregulates *PINK1* expression in macrophages and that *PINK1* knockdown results in decreased cytokine production and attenuated IRF3 and NF-κB activation upon viral infection [182].

Parasitic and fungal infections may also trigger an immune response, leading to inflammation in PD: Some studies have found a higher prevalence of antibodies against *Toxoplasma gondii* in individuals with PD compared to healthy individuals [183]. Additionally, individuals with PD have been observed to have a higher prevalence of Malassezia on their skin compared to healthy individuals [184]. Conversely, some studies have suggested that exposure to certain types of helminths may have a protective effect against autoimmune diseases and inflammatory conditions, including PD [185].

In conclusion, infections profoundly influence immune responses in both the periphery and CNS, and have been suggested to contribute to the development and/or progression of PD. It is important to note that while there is some evidence suggesting a potential link between infections, neuroinflammation, and PD, it currently remains unclear whether inflammation is a consequence of neural cell death or whether different inflammatory pathways contribute to cell death in PD.

## 9. Clinical trials using anti-inflammatory drugs

Despite strong preclinical evidence linking immune activation to PD pathogenesis and progression, large-scale clinical trials on immunomodulatory treatments have failed to be successful (for review, see ref. [186]). Although some recent early-phase studies suggest promising effects, validation in larger trials is the next crucial step. These include for instance medications such as Lixisenatide, a GLP-1 agonist [187], Montelukast, a cysteinyl leukotriene receptor type 1 antagonist (phase-II trial completed: EUCTR2020-000148-76-SE [188] phase-II ongoing: EudraCT number 2023-504278-39-00 [189], and NE3107, a brain-penetrant compound that inhibits inflammatory cascades in macrophages (phase-II trial completed: NCT05083260,[189, 190]). Additionally, several other trials have just been completed or are currently in progress, and their results are eagerly anticipated [186]. Furthermore, it is likely that other compounds demonstrating effects in preclinical studies will be tested in clinical trials in the future. For example, cyclosporin A has shown beneficial effects in preclinical PD models, including human cell cultures [191] and rodent models [191, 192] but clinical trials are pending. Several factors may account for the limited success of clinical trials to date: First, PD is a complex and heterogeneous disease, with inflammation possibly playing a varying role among patients, making it difficult to identify responders. Despite this, most trials have not incorporated biomarker-based approaches to identify patients who may have benefited from treatment among the group of treated patients [186]. Second, timing is crucial—clinical trials often include patients with established PD, where neurodegeneration may already be too advanced for anti-inflammatory therapies to have a disease-modifying effect. Additionally, many studies have tested repurposed anti-inflammatory drugs, which may not specifically target PD-related inflammatory pathways. Furthermore, given the prolonged clinical recovery times seen in other CNS conditions, treatment durations in some trials may have been too short to observe relevant clinical effects. Future trials may improve outcomes by incorporating biomarker-driven patient selection and monitoring, initiating interventions as early as possible, prolonging treatment times, increasing patient numbers, and exploring novel or combination therapies.

## 10. Upcoming techniques

For about two decades, starting with the advent of genome-wide genotyping, data generation and analysis in both epidemiological studies as well as laboratory experiments has begun to shift more and more from targeted to large-scale analyses targeting (nearly) the entire respective molecular domain, not only for genomics and transcriptomics, but also to epigenomics, metabolomics, lipidomics, and proteomics using next-generation sequencing and Mass Spectometry techniques [193]. Single cell next-generation sequencing becomes more and more feasible also on a large scale [194]. Affinity-based proteomic methods such as those using Olink® and SomaScan® technologies currently measure several thousand proteins at once, which have been preselected based on functional considerations and include many immunologically relevant proteins. We have just started to explore the many possibilities of jointly analyzing such omics data also in the immunological field in PD. A few examples of transcriptomic studies were described above. Ultimately, leveraging high-throughput molecular omics techniques and their combined analyses will mark the next important step towards understanding the immune responses that drive and underlie Parkinson's disease pathology.

## 11. Conclusion

Taken together, a substantial body of evidence suggests that the immune system contributes to PD pathogenesis and progression, alongside mitochondrial dysfunction, oxidative stress, and α-synuclein aggregation. Specifically, microglia and astrocytes activated by α-synuclein and oxidative stress contribute to a neurotoxic environment, fostering a self-perpetuating cycle of neurodegeneration, oxidative damage, and chronic inflammation. Additionally, the infiltration of activated peripheral CD4+ and CD8+ T cells into the brain through a compromised blood-brain barrier exacerbates neuroinflammation and neuronal loss. Some studies also indicate that infections may act as potential triggers or contributors to PD.

However, findings from human immunological studies in PD remain inconsistent and sometimes contradictory. This variability arises, at least in part, from several key factors: small sample sizes leading to reduced statistical power and false positives, heterogeneous patient populations (e.g., early-onset vs. late-onset, early vs. advanced disease stages), and a lack of consideration for critical demographic, lifestyle, and clinical variables (such as disease duration, treatment effects, sex, age at onset, age at examination, lifestyle factors such as smoking status, and comorbidities) as well as methodological aspects with lack of standardization. For instance, for flow cytometry, it is common for each study to employ its own combination of markers and fluorochromes, even when examining comparable cell types. Sample handling, instrument type and configuration, gating and analysis strategies as well as data reporting methods are other key points that impact the results and their interpretation [195]. To ensure comparability between studies, standardized procedures must be established to improve reproducibility and validity. The same applies to emerging *omics*-based approaches, including the promising field of single-cell *omics* technologies, which is likely to revolutionize the field in the coming years.

Elucidating the interplay between chronic inflammation, mitochondrial dysfunction, and α-synuclein aggregation in PD is crucial not only for understanding pathophysiology but also for identifying novel therapeutic strategies. Potential interventions include targeting neuroinflammation (e.g., NLRP3 inflammasome inhibitors or immunomodulatory therapies), enhancing mitophagy through PINK1/parkin pathway activators to clear dysfunctional mitochondria, or reducing α-synuclein aggregation via immunotherapy. In these clinical trials, advanced immunological methodologies, single-cell multi-omics technologies, and PET imaging may be used to monitor individual treatment responses. Given the self-perpetuating nature of neuroinflammation and neurodegeneration in PD, along with its prolonged prodromal phase, early intervention is critical. This underscores the urgent need for reliable biomarkers to detect PD in its preclinical stages, enabling the development of preventive or early therapeutic strategies. Ongoing studies (e.g., ref. [196]) are currently investigating whether immunological signatures and biomarkers could facilitate early PD detection, paving the way for more effective interventions.
